# The mRNA and Proteins Expression Levels Analysis of TC-1 Cells Immune Response to H9N2 Avian Influenza Virus

**DOI:** 10.3389/fmicb.2016.01039

**Published:** 2016-06-30

**Authors:** Jiyuan Liu, Ning Li, Dan Meng, Mengchan Hao, Liangmeng Wei, Tongjie Chai

**Affiliations:** ^1^College of Animal Science and Veterinary Medicine, Shandong Agricultural UniversityTai’an, China; ^2^Sino-German Cooperative Research Centre for Zoonosis of Animal Origin Shandong ProvinceTai’an, China; ^3^Collaborative Innovation Centre for the Origin and Control of Emerging Infectious Diseases of Taishan Medical CollegeTai’an, China

**Keywords:** H9N2 avian influenza virus, TC-1 cells, pattern recognition receptors, cytokines, chemokines

## Abstract

Since 1994, the H9N2 avian influenza virus (AIV) has spread widely in mainland China, causing great economic losses to the poultry industry there. Subsequently, it was found that the H9N2 AIV had the ability to infect mammals, which gave rise to great panic. In order to investigate the immune response of a host infected with H9N2 AIV, TC-1 cells were set as a model in this research. Quantitative real-time polymerase chain reaction and enzyme-linked immunosorbent assay methods were used to study the expression changes of pattern recognition receptors (PRRs), inflammatory cytokines, and chemokines in AIV-infected TC-1 cells. Our research found that TC-1 cells had similar susceptibility to both CK/SD/w3 (A/Chicken/Shandong/W3/2012) and CK/SD/w4 (A/Chicken/Shandong/W4/2012) H9N2 isolates, while the CK/SD/w3 isolate had a stronger capability of replication in the TC-1 cells. At the same time, the expression of PRRs (melanoma differentiation-associated gene 5, MDA-5), cytokines [interleukin (IL)-1β and IL-6], and chemokines [regulated on activation, normal T cell expressed and secreted (RANTES) and interferon-γ-induced protein-10 kDa (IP-10)] were significantly up-regulated. These results indicated that MDA-5, IL-1β, IL-6, RANTES, and IP-10 might play important roles in the host immune response to H9N2 AIV infection. This study provided useful information for further understanding the interaction between H9N2 virus infection and host immunity, and had certain guiding significance for the prevention and treatment of this disease.

## Introduction

H9N2 avian influenza viruses (AIVs), which belong to the Orthomyxoviridae family, have been circulating worldwide in many avian species and have led to great economic losses (Li et al., 2005; Ducatez et al., 2008; Nagarajan et al., 2009). What’s more, human cases of H9N2 AIV infection have been reported in Hong Kong and mainland China since the late 1990s (Peiris et al., 1999; Butt et al., 2005; Cheng et al., 2011). Subsequently, specific antibodies to H9N2 AIV were detected in poultry workers and patients (Guo et al., 1999; Guan et al., 2000; Lin et al., 2000). These findings pointed to a possibility that H9N2 AIV could infect humans directly, which caused great public concern.

Although H9N2 AIV is low-pathogenic type virus, causing only asymptomatic infection or a mild disease in poultry populations (WHO, 2004), research has proven that six internal genes of the highly pathogenic type AIV (HPAIV)—such as H7N9 and H10N8—are closely related to the H9N2 virus (Gao et al., 2013; Kageyama et al., 2013; Chen et al., 2014; Neumann et al., 2014); H9N2 AIV is thought of as the incubator for many HPAIVs (Liu et al., 2014). Hence, more and more researchers have paid attention to H9N2 AIV in recent years (Maines et al., 2008).

Innate immunity is the first barrier system to the invading pathogens. The host pattern recognition receptors (PRRs) recognize the pathogen-associated molecular patterns (PAMPs), leading to activating the innate immunity response (Kawai and Akira, 2009; Palm and Medzhitov, 2009; Takeuchi and Akira, 2009). PRRs mainly consist of Toll-like receptors (TLRs) and RIG-like receptors (RLRs). TLRs (TLR-3/TLR-7/TLR-8) recognize the PAMPs at the cell surface (Akira et al., 2006) and RLRs function in the cytosol. The melanoma differentiation-associated gene 5 (MDA-5) belongs to the RLR group, which recognize viral RNA in the cytosol and start innate immunity responses, characterized by the induction of interferons (IFNs), IFN-stimulated genes, and proinflammatory cytokines (Yoneyama et al., 2004; Kato et al., 2006; Yoneyama and Fujita, 2007, 2009; Jensen and Thomsen, 2012).

Lung epithelial cells, which were seen as the key target cells of influenza viruses, play a pivotal role in the initial response to influenza virus (Matsukura et al., 1996; Seo and Webster, 2002; Raj et al., 2011). In previous studies, we detected expression change in the mRNA and protein levels of six cytokines [IFN-β, IFN-γ, tumor necrosis factor (TNF)-α, interleukin (IL)-1β, IL-6, and IL-10] and five chemokines [IFN-γ-induced protein-10 kDa (IP-10), chemokine ligand-5 (CCL-5), monocyte chemoattractant protein-1, macrophage inflammatory protein-1 alpha, and IL-8] in the lungs of H9N2 AIV-infected mice (Huang et al., 2013). However, the immune response of TC-1 cells after infection with H9N2 AIV was unknown.

To complete the knowledge of the mechanism of pathogenicity and immunity, TC-1 cells were infected by CK/SD/w3 (A/Chicken/Shandong/W3/2012) and CK/SD/w4 (A/Chicken/Shandong/W4/2012) viruses at a multiplicity of infection (MOI) of 1, respectively. Then, quantitative real-time polymerase chain reaction (qRT-PCR) and enzyme-linked immunosorbent assay (ELISA) methods were used to detect the content of three PRRs (TLR-3, TLR-7, and MDA-5) and four cytokines/chemokines (IL-1β, IL-6, IP-10, and CCL-5) in mRNA levels and protein levels. The results were useful to understand the interaction between H9N2 virus infection and host immunity *in vitro*.

## Materials and Methods

### Virus and Cell Cultures

The H9N2 viruses, CK/SD/W3 (Huang et al., 2013) and CK/SD/W4, belong to the BJ94-like lineage. The viruses were passaged in 10-day-old specific pathogen-free (SPF) chicken embryos. The 50% tissue-culture-infective dose (TCID_50_) was calculated using the method of Reed and Muench (1938).

TC-1 cells were derived from mouse lung epithelial, which we purchased from the China Center for Type Culture Collection, where they were grown as monolayers in RPMI-1640 medium (Gibco) supplemented with 10% fetal bovine serum (FBS), 100 U/ml penicillin, and 100 μg/ml streptomycin at 37°C in a 5% CO_2_ incubator. Madin–Darby canine kidney (MDCK) cells were used for growing stocks of virus isolates. MDCK cells were grown and maintained in Dulbecco’s Minimal Essential Medium (Gibco) containing 10% FBS, 100 U/ml penicillin, and 100 μg/ml streptomycin.

### Infection of Cell Culture with H9N2 AIV

TC-1 cells were grown in T25 tissue culture flasks for the inoculation of virus isolate (MOI = 1). After 2 h of adsorption, the virus was removed and 5 ml of fresh RPMI-1640 media, supplemented with 1% FBS, 100 U/ml penicillin, 100 μg/ml streptomycin, and 2 μg/ml L-1-tosylamide-2-phenylethyl chloromethyl ketone-treated trypsin, was added. The control groups were infected with the allantoic fluid of a healthy SPF chick embryo.

### Immunofluorescence

TC-1 cells were fixed with an acetone and ethanol solution (acetone:ethanol = 3:2) for 5 min, then washed with phosphate-buffered saline (PBS). Air-dried cells were incubated with an anti-hemagglutinin (HA) monoclonal antibody at a 1:500 dilution, at 37°C, for 1 h. After three washes with PBS, cells were incubated with fluorescein isothiocyanate (FITC)-conjugated goat anti-mouse IgG at a 1:500 dilution, at 37°C, for 45 min. After three washes with PBS, cells were air-dried, and observed with a Nikon inverted fluorescence microscope.

### Cytokine/Chemokine and PRRs mRNA Expression Profile

Infected TC-1 cell cultures were harvested at various time points and total RNA was extracted using the TRIzol reagent (Invitrogen). Total RNA was reversely transcripted to cDNA using the PrimeScript^TM^ RT Reagent Kit with gDNA Eraser (TaKaRa), and quantified by real-time PCR. qRT-PCR reactions were performed in triplicate using the SYBR Green PCR Master Mix (Applied Biosystems). The expression of the β-actin gene was also quantified in a similar way for normalization. The data of infected and uninfected gene expression were analyzed by the 2^-ΔΔCt^ method.

### Quantification of Cytokine/Chemokine PRRs Protein Expression

Cell culture medium supernatant was collected at 6, 12, 18, 24, 36, and 48 h post-infection (hpi). We used a capture ELISA reagent kit (PBL Biomedical Laboratories, Piscataway, NJ, USA) to measure levels of secreted cytokine/chemokine and PRR protein in the culture supernatant of uninfected and H9N2-infected TC-1 cells, according to the manufacturer’s protocol. Data were normalized according to the total protein quantities of the samples.

### Statistical Analysis

The 2^-ΔΔCt^ method and standard curve were taken to process data of qRT-PCR and ELISA, respectively. Statistical significance was calculated by the Statistical Analysis System. *P* < 0.05 was considered to be significant.

## Results

### Susceptibility of TC-1 Cells to Avian H9N2 Virus

**FIGURE 1:**
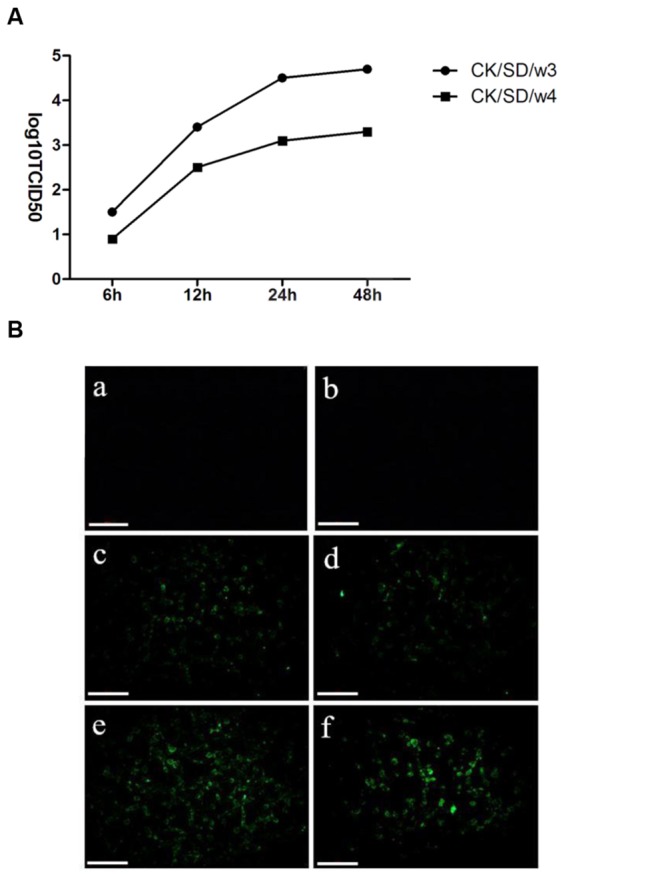
**Kinetics of replication of CK/SD/w3 and CK/SD/w4 in TC-1 cells. (A)** Infectious viruses from TC-1 cultures were harvest at 6, 12, 24, and 48 hpi, respectively, and titrated at MDCK cells; **(B)** CK/SD/w3 and CK/SD/w4 infection in TC-1 cells. CK/SD/w3 influenza virus (left); CK/SD/w4 influenza virus (right). Cells were infected with the viruses for 6 and 12 h, respectively, and stained with an fluorescein isothiocyanate (FITC)-anti-influenza A hemagglutinin (HA) antibody. Subsequently fluorescence images were taken (magnification, ×200; scale bars: 20 μm): (a,b) uninfected cells; (c,d) 6 h after infection; (e,f) 12 h after infection.

To detect the differences of viral infectivity, viral replication in TC-1 cells was examined. The infectious viruses were diluted by culture media, and then titrated on MDCK cells. The results revealed that CK/SD/w3 had a slightly stronger replication capacity than CK/SD/w4 in infected TC-1 cells (**Figure 1A**).

To examine the susceptibility of TC-1 cells to the H9N2 viruses, we infected TC-1 cells with the H9N2 viruses at an MOI = 1. Then, the cells stained by FITC-anti-influenza HA antibody. As shown in **Figure 1B**, the cells infected with CK/SD/w3 became positive by FITC-anti-influenza HA antibody staining at 6 hpi, While sporadic cell became positive at 6 hpi in CK/SD/w4 H9N2 infection. CK/SD/w3 and CK/SD/w4 have the similar pattern of infection. However, there were more cells stained by FITC-anti-influenza HA antibody in CK/SD/w3 infection at 12 hpi. The results suggested that TC-1 cells were more sensitive to CK/SD/w3 than CK/SD/w4 (**Figure 1B**).

### PRRs mRNA Expression

**FIGURE 2:**
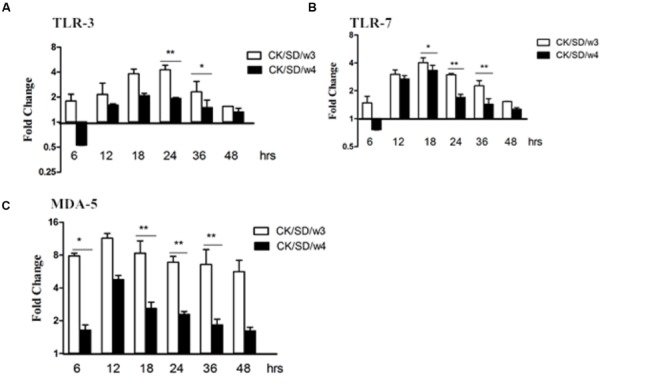
**TLR-3, TLR-7, and MDA-5 mRNA levels at various time points post-infection.** TC-1 cells were infected with CK/SD/w3 and CK/SD/w4 at MOI = 1. qRT-PCR were used to examine the mRNA levels and fold-changes was calculated by 2^-ΔΔCt^ method as compared with non-infection cell control and using endogenous β-actin mRNA level for normalization. **(A)** The fold change of TLR-3 in mRNA levels; **(B)** The fold change of TLR-7 in mRNA levels; **(C)** The fold change of MDA-5 in mRNA levels. The data was shown the as mean ± SE from three sets of independent experiments. ^∗^*P* < 0.05; ^∗∗^*P* < 0.01.

To determine which PRRs were involved in H9N2-infected airway epithelial cultures, we examined the expression levels of a few pathogen sensor receptors. We focused on TLR-3 and TLR-7, which are involved in responses to RNA viruses, as well as MDA-5, which responds to double-stranded RNA (dsRNA). Results indicated that there were moderate increases in TLR-3 and TLR-7, especially at 18 hpi. The concentration of TLR-3 increased up to the peak of fourfold at 24 hpi (*P* < 0.01; **Figure 2A**), and TLR-7 increased up to fourfold at 18 hpi (*P* < 0.05; **Figure 2B**), after both were infected with CK/SD/w3.

Moreover, there was obvious up-regulation of MDA-5 after infection with both AIV isolates. What is noteworthy is that MDA-5 was increased up to 7.8- and 11.4-fold at 6 and 12 hpi (*P* < 0.05; **Figure 2C**), respectively, in the samples infected with CK/SD/w3 (**Figure 2C**). In the CK/SD/w4 infection test, expression levels reached their peak at 12 hpi (a change of about 4.8-fold, *P* > 0.05; **Figure 2C**), which was lower than in the CK/SD/w3 infection test. These results indicated that MDA-5 may play a more important role than TLR-3 and TLR-7 at the early stage of H9N2 AIV infection.

### Cytokine/Chemokine mRNA Expression

**FIGURE 3:**
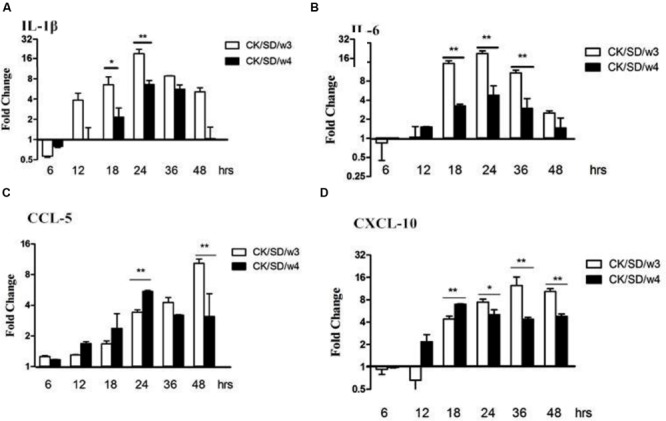
**Cytokine and chemokine mRNA levels at various time points post-infection.** TC-1 cells were infected with CK/SD/w3 and CK/SD/w4 at MOI = 1. qRT-PCR were used to quantify the mRNA levels and fold-changes was calculated by 2^-ΔΔCt^ method as compared with non-infection cell control and using endogenous actin mRNA level for normalization. **(A)** The fold change of IL-1β in mRNA levels; **(B)** The fold change of IL-6 in mRNA levels; **(C)** The fold change of CCL-5 in mRNA levels; **(D)** The fold change of chemokine (C-X-C motif) ligand-10 (CXCL-10) in mRNA levels. The data was shown as the mean ± SE from three sets of independent experiments. ^∗^*P* < 0.05; ^∗∗^*P* < 0.01.

After both AIV isolates infection, the mRNA expression levels of cytokines/chemokines were examined. Results indicated that CK/SD/w3 showed a higher capacity in inducing IL-1β and IL-6 compared with that of CK/SD/w4 (**Figures 3A,B**). By qRT-PCR, we found that there were no obvious up-regulations in the expressions of IL-1β and IL-6 during the early phase infection of CK/SD/w3 and CK/SD/w4. However, there were marked increases in the expression of IL-1β and IL-6 post-infection with CK/SD/w3 at 12 hpi (*P* > 0.05; **Figure 3A**) and 18 hpi (*P* < 0.05; **Figure 3A**), respectively, reaching the highest expression at 24 hpi (IL-1β at 19-fold and IL-6 at 21-fold, *P* < 0.01; **Figures 3A,B**).

On the other hand, chemokines CCL-5 and chemokine (C-X-C motif) ligand-10 (CXCL-10) moderately increased post-virus infection. CK/SD/w4 showed a higher capacity in inducing chemokines compared with that of CK/SD/w3 at the early stage of infection. The concentrations of CCL-5 in the CK/SD/w4 infection were up-regulated to fivefold at 24 hpi (*P* < 0.01; **Figure 3C**), whereas CXCL-10 was increased up to sevenfold at 18 hpi (*P* < 0.01; **Figure 3D**). In contrast, CK/SD/w3 has more capacity in inducing CCL-5 and CXCL-10 at the later stage of infection. Our data indicated that concentrations of CCL-5 and CXCL-10 were up-regulated up to 10- and 12-fold at 48 and 36 hpi, respectively (*P* < 0.01; **Figures 3C,D**).

### PRRs Protein Profiles Follow Infection

**FIGURE 4:**
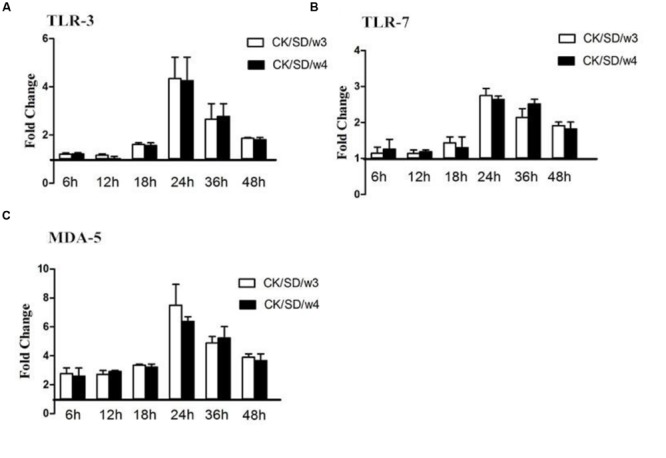
**TLR-3, TLR-7, and MDA-5 protein levels at various time points post-infection.** TC-1 cells were infected with CK/SD/w3 and CK/SD/w4. The supernatants were collected at 6, 12, 18, 24, 36, and 48 h post-infection and assayed by ELISA. Fold-change was calculated by compared with non-infection control supernatants. **(A)** The fold change of TLR-3 in protein levels; **(B)** The fold change of TLR-7 in protein levels; **(C)** The fold change of MDA-5 in protein levels. The data was shown as the mean ± SE from three sets of independent experiments. ^∗^*P* < 0.05; ^∗∗^*P* < 0.01.

At the same time, the concentration of PRR protein was detected by the ELISA kit. The actual concentrations of PRRs were displayed in **Table 1**. There was a moderate up-regulation for TLR-3 and TLR-7 in CK/SD/w3 and CK/SD/w4 infection. In CK/SD/w3-infected cells, TLR-3 and TLR-7 were induced to a high level at 24 hpi (4.3- and 2.7-fold, respectively; *P* > 0.05; **Figures 4A,B**). The TLR-7 concentration remained at a relatively lower-fold change than that of CK/SD/w3 throughout the time course examined. These results correlated with the TLR-3 and TLR-7 mRNA levels, except for some deviations.

**Table 1:** The actual concentrations of PRRs, cytokines, and chemokines (pg/ml).

	6 h	12 h	18 h	24 h	36 h	48 h
**Control**					
TLR-3	12.11 ± 1.59	12.02 ± 1.93	13.39 ± 1.74	14.97 ± 2.35	10.54 ± 0.97	11.09 ± 1.23
TLR-7	7.30 ± 1.97	6.48 ± 2.45	8.18 ± 1.47	7.96 ± 1.93	6.55 ± 0.76	7.35 ± 2.17
MDA-5	1.49 ± 0.98	2.79 ± 0.74	4.55 ± 1.35	3.79 ± 1.56	1.94 ± 1.74	1.43 ± 1.11
IL-1β	12.57 ± 2.38	10.82 ± 1.94	12.81 ± 2.38	9.05 ± 0.76	11.34 ± 3.43	15.10 ± 4.03
IL-6	7.15 ± 2.58	7.42 ± 1.39	10.05 ± 2.34	6.62 ± 1.52	6.37 ± 1.01	8.18 ± 2.43
CCL-5	2.24 ± 0.97	3.01 ± 1.03	3.04 ± 1.04	2.98 ± 0.76	2.43 ± 0.83	2.25 ± 0.99
CXCL-10	4.82 ± 1.43	5.24 ± 0.75	6.57 ± 1.81	6.22 ± 1.24	4.94 ± 1.13	5.48 ± 1.54
**CK/SD/w3**					
TLR-3	15.27 ± 2.38	14.76 ± 4.25	18.69 ± 3.33	41.85 ± 8.54	24.24 ± 6.73	22.19 ± 5.53
TLR-7	9.60 ± 3.26	7.60 ± 1.43	9.24 ± 2.55	21.54 ± 4.36	13.83 ± 2.13	13.24 ± 1.33
MDA-5	3.52 ± 0.79	6.98 ± 1.35	13.65 ± 3.33	13.33 ± 1.45	7.97 ± 1.34	5.46 ± 1.34
IL-1β	10.03 ± 2.33	15.11 ± 2.41	22.41 ± 4.44^∗∗^	25.34 ± 3.37	31.77 ± 5.53	21.14 ± 1.11
IL-6	7.51 ± 1.04	7.33 ± 1.87	11.63 ± 2.01	21.19 ± 3.33^∗^	18.47 ± 2.57	16.36 ± 3.49
CCL-5	17.84 ± 3.42	31.44 ± 6.31	33.84 ± 5.48	79.04 ± 12.55	73.04 ± 9.37	85.84 ± 15.35
CXCL-10	7.97 ± 1.34	7.69 ± 2.54	18.11 ± 2.77	40.43 ± 8.34	37.56 ± 5.44	26.05 ± 3.99
**CK/SD/w4**					
TLR-3	15.35 ± 3.34	11.34 ± 2.13	17.49 ± 3.35	39.80 ± 7.13	27.32 ± 5.54	20.74 ± 4.83
TLR-7	11.18 ± 2.38	8.30 ± 1.56	7.30 ± 0.89	19.95 ± 3.34	19.30 ± 4.05	11.89 ± 1.83
MDA-5	3.06 ± 0.89	7.23 ± 1.35	12.34 ± 2.34	10.01 ± 2.54	8.97 ± 1.11	4.79 ± 0.97
IL-1β	9.87 ± 1.32	12.73 ± 3.43	19.39 ± 4.44	30.51 ± 5.54	32.49 ± 4.88	18.20 ± 2.79
IL-6	5.93 ± 1.30	9.78 ± 2.13	11.19 ± 2.22	19.17 ± 3.54	16.45 ± 3.87	11.63 ± 2.13
CCL-5	7.84 ± 1.43	12.64 ± 2.22	25.04 ± 4.45	71.84 ± 8.38	55.04 ± 4.34	71.04 ± 7.83
CXCL-10	4.13 ± 0.37	2.35 ± 0.97	12.76 ± 3.34	34.27 ± 4.43	30.84 ± 4.43	23.72 ± 5.79

*The data was shown as the mean ± SE (^∗^*P* < 0.05; ^∗∗^*P* < 0.01, *n* = 3).*

Both CK/SD/w3 and CK/SD/w4 could induce the expression of MDA-5 from the early stage of infection; the fold-change peaked at 24 hpi (a 7.5- and 6.4-fold change, respectively; *P* > 0.05; **Figure 4C**), then increased gradually. However, the concentration of MDA-5 reached peak at 18 hpi (**Table 1**). The results indicated that MDA-5 played a key role in H9N2 AIV infections.

### Cytokine/Chemokine Protein Profiles Following Infection

**FIGURE 5:**
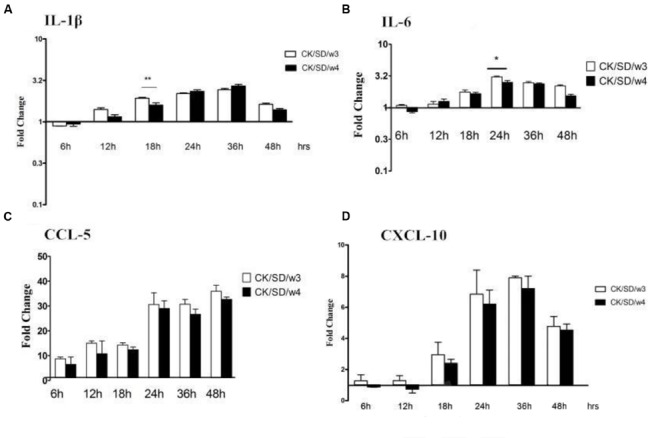
**Cytokine and chemokine protein levels at various time points post-infection.** TC-1 cells were infected with CK/SD/w3 and CK/SD/w4. The supernatants were collected at 6, 12, 18, 24, 36, and 48 h post-infection and assayed by ELISA. Fold-change were calculated by compared with non-infection control supernatants. **(A)** The fold change of IL-1β in protein levels; **(B)** The fold change of IL-6 in protein levels; **(C)** The fold change of CCL-5 in protein levels; **(D)** The fold change of CXCL-10 in protein levels. The data was shown as the mean ± SE from three sets of independent experiments. ^∗^*P* < 0.05; ^∗∗^*P* < 0.01.

To verify whether changes at the mRNA level were translated to the protein level, the protein concentrations of cytokines/chemokines in cell culture supernatants were measured (**Table 1**). As indicated in **Figure 5**, the data showed that TC-1 cells secreted high amounts of IL-1β, IL-6, CCL-5, and CXCL-10 in response to influenza virus infections. In CK/SD/w3-infected cells, CCL-5 and CXCL-10 were induced to high levels at 36 and 24 hpi (34- and 9-fold, respectively; *P* > 0.05; **Figures 5C,D**). Otherwise, CK/SD/w4 induced a high level of IL-1β at 36 hpi (about 2.6-fold, **Figure 5A**). The level of IL-6 was similar to IL-1β. The results showed that induction of chemokine was more prominent throughout the time course examined in the CK/SD/w3 infection. CK/SD/w3 showed 34- and 9-folds (*P* > 0.05; **Figures 5C,D**) of induction for CCL-5 and CXCL-10, respectively, which was higher than the induction of CK/SD/w4.

The highest levels of induction for IL-1β and IL-6 were observed at 36 and 24 hpi, respectively. But the concentration of IL-1β reached the peak (about 2.6-fold, *P* > 0.05; **Figure 5A**) in CK/SD/w4 infections, and the IL-6 in CK/SD/w3 infections (about 2.8-fold, *P* < 0.05; **Figure 5B**). In general, CK/SD/w3 showed a higher capacity in inducing cytokine/chemokine as compared with that of CK/SD/w4. What’s more, the cytokine/chemokine protein levels correlated with the corresponding mRNA transcription levels, except for IL-1β.

## Discussion

Lung epithelial cells were the target cell of the influenza virus (Matsukura et al., 1996; Seo and Webster, 2002). In previous studies, researchers were focus on the pathogenesis of influenza viruses, and there are rare studies on the immune mechanism of influenza virus infection.

CK/SD/w3 and CK/SD/w4 were isolated from chickens in Shandong, China, which belonged to the BJ-94 lineage. The previous research showed that there was a new glycosylation site (Asn-Cys-Ser) at the 313 amino acid residue in the HA protein of CK/SD/w3, and a new glycosylation site (Asn-Arg-Thr) at the 218 amino acid residue in the HA protein of CK/SD/w4 (Huang et al., 2014). In addition, it has been demonstrated that CK/SD/w3 changed the valine to alanine at the 198 amino acid residue, and CK/SD/w3-infected mice efficiently while CK/SD/w4 did not (Huang et al., 2014), which indicated that the change of HA receptor binding site also influence the virulence of H9N2 AIVs. Although both CK/SD/w3 and CK/SD/w4 can replicate in mammalian cells, the ability of replication and pathogenicity were different, which indicated that the potential glycosylation site of the HA protein may be one of the factors that affected the virulence of influenza viruses. Furthermore, the change of glycosylation sites may be the reason for CK/SD/w3 and CK/SD/w4 replication in mammalian cells (Huang et al., 2014). It was the reason CK/SD/w3 and CK/SD/w4 have differences in replication kinetics and infection abilities when it comes to TC-1 cells.

Research showed that both membrane-bound TLR and cytosolic retinoic acid-inducible gene 1 (RIG-1)-like receptors were involved in the response to the influenza virus in human airway epithelial cells. During the replication periods of the influenza virus, there were three kinds of viral RNA methods—ssRNA, dsRNA, and a self-paired double-stranded structure in the host, which could be recognized by TLR-3, TLR-7, and MDA-5. The downstream nuclear factor-κB (NF-κB) and interferon regulatory factors (IRFs) can be activated by TLR-3, TLR-7, and MDA-5, and then the host produces cytokines and chemokines, such as IL-6 and CCL-5. The current study results indicated that, both in mRNA and protein levels, MDA-5 was significantly up-regulated post-infection with CK/SD/w3 and CK/SD/w4 (*P* < 0.05; **Figures 2C and 4C**). TLR-3 and TLR-7 had the significantly up-regulation at 24 hpi (**Figures 2A,B and 4A,B**), indicating that MDA-5, TLR-3, and TLR-7 were involved in the host immune response after CK/SD/w3 and CK/SD/w4 infected the TC-1 cells. It is worth noting that MDA-5 was highly expressed in the whole process, but TLR-3 and TLR-7 were only significantly up-regulated at 24 hpi in mRNA and protein levels (**Figures 2** and **4**). This phenomenon was similar to the previous study (Zheng et al., 2011) and indicated that MDA-5 was an important PRR in the host anti-viral immune response.

Cytokines and chemokines are important in the process of resistance and clearance of the virus. IL-1β was the main form of IL-1 secretion (Leon, 2002). IL-1β, as a proinflammatory cytokine, mediates the host response to infection through both direct and indirect means. With tissue damage, IL-1β increases in concentration at the site of inflammation. It can also induce the release of inflammatory cytokines, such as IL-6, IL-8, and TNF-α, then promote the inflammation response (Zheng and Jiang, 2010). In this study, we found that the mRNA contents of IL-1β and IL-6 were significantly up-regulated in CK/SD/w3- and CK/SD/w4-infected cells. This occurred 6.5- and 12.1-fold (*P* < 0.01; **Figure 3A**) in the CK/SD/w3 infection test, and 2.1- and 3.2-folds (*P* < 0.01; **Figure 3B**) in the CK/SD/w4 infection test, respectively, peaking at 24 hpi (**Figures 3A,B**). The contents of IL-1β and IL-6 were also increased in the cell supernatant (**Figures 5A,B**). These results indicated that CK/SD/w3 and CK/SD/w4 could cause a more intense inflammatory reaction in TC-1 cells. In previous studies (Lam et al., 2010; Zheng et al., 2011), the contents of IL-6 and IL-1β were not significantly changed after H9N2 AIV infection. This may be due to the use of different strains and different experimental cells.

CCL-5 and CXCL-10 are the major chemokines in inflammatory reaction. They play an important role in the directional trend to white blood cells to the damaged parts of the respiratory tract. In this study, both CXCL-10 and CCL-5 were significantly up-regulated at mRNA levels, which were similar to previous research (Zhou et al., 2006; Zheng et al., 2011). What’s more, the up-regulation of protein levels was similar to that of mRNA levels.

In summary, CK/SD/w3 and CK/SD/w4 have differences in virulence in TC-1 cells. The expressions of PRRs and cytokines/chemokines were also different, but both CK/SD/w3 and CK/SD/w4 could induce MDA-5, regulated on activation, normal T cell expressed and secreted (RANTES), and IP-10, and significantly increase in mRNA and protein levels. These results indicated that MDA-5, RANTES, and IP-10 played key roles in the immunity response to H9N2 AIVs in TC-1 cells. A cell body had no complex regulation mechanism, and the immune system that was incomplete. So the immune response of infected TC-1 cells was unique to whole body. But, our study will provide a helpful reference to understanding of the host immunity response to AIVs.

## Author Contributions

JL and NL designed and conducted the study, performed most of the experiments, and wrote the manuscript. DM performed the calculation, MH performed the biological experiments. LW and TC designed the study and wrote the manuscript.

## Conflict of Interest Statement

The authors declare that the research was conducted in the absence of any commercial or financial relationships that could be construed as a potential conflict of interest.
